# Distribution of *Anopheles daciae* and other *Anopheles maculipennis* complex species in Serbia

**DOI:** 10.1007/s00436-018-6028-y

**Published:** 2018-08-28

**Authors:** Mihaela Kavran, Marija Zgomba, Thomas Weitzel, Dusan Petric, Christina Manz, Norbert Becker

**Affiliations:** 1 Faculty of Agriculture, University of Novi Sad, Trg Dositeja Obradovića 8, Novi Sad, 21000 Serbia; 2 German Mosquito Control Association (KABS), Georg-Peter-Süß-Str. 3, 67346 Speyer, Germany; 3 Universität Karlsruhe (TH), Kaiserstraße 12, 76131 Karlsruhe, Baden-Württemberg Germany

**Keywords:** *Anopheles maculipennis* complex, Malaria, *Anopheles daciae*, ITS2

## Abstract

Malaria is one of the most severe health problems facing the world today. Until the mid-twentieth century, Europe was an endemic area of malaria, with the Balkan countries being heavily infested. Sibling species belonging to the *Anopheles maculipennis* complex are well-known as effective vectors of *Plasmodium* in Europe*.* A vast number of human malaria cases in the past in the former Yugoslavia territory have stressed the significance of *An. maculipennis* complex species as primary and secondary vectors. Therefore, the present study evaluates the species composition, geographic distribution and abundance of these malaria vector species. Mosquitoes were collected in the northern Serbian province of Vojvodina and analysed by PCR-RFLP, multiplex PCR and sequencing of the ITS2 intron of genomic rDNA. Four sibling species of the *An. maculipennis* complex were identified. Both larvae and adults of the recently described species *An. daciae* were identified for the first time in Serbia. In 250 larval samples, 109 (44%) *An. messeae*, 90 (36%) *An. maculipennis* s.s., 33 (13%) *An. daciae* and 18 (7%) *An. atroparvus* were identified*.* In adult collections, 81 (47%) *An. messeae*, 55 (32%) *An. daciae*, 33 (19%) *An. maculipennis* s.s., and 3 (2%) *An. atroparvus* were recorded. The most abundant species in Vojvodina was *An. messeae*, whereas *An. atroparvus* was confirmed a rare species in all parts. Since this species is a potentially, highly competent malarial vector, low population density could be crucial to prevent a new establishment of endemic malaria transmission in Serbia.

## Introduction

The discovery that mosquitoes can transmit microfilariae and malarial protozoa at the end of the nineteenth century initiated the collection, naming and classification of *Anopheles* species after the genus was introduced by Johann Wilhelm Meigen in 1818. *Anopheles maculipennis* was first recognised by van Thiel (1927) to be a complex of sibling species or races, since larvae, pupae and adults are mostly indistinguishable from each other by morphological characters. Eleven species of the *An. maculipennis* complex (AMC) are formally considered in the Palaearctic region: *Anopheles artemievi* Gordeyev, Zvantsov, Goryacheva, Shaikevich and Yezhov, 2005; *Anopheles atroparvus* van Thiel, 1927; *Anopheles beklemishevi* Stegnii & Kabanova (1976); *Anopheles daciae* Linton, Nicolescu and Harbach, 2004; *Anopheles labranchiae* Falleroni, 1926; *Anopheles maculipennis* s.s. Meigen, 1818; *Anopheles martinius* Shinagarev, 1926; *Anopheles melanoon* Hackett, 1934; *Anopheles messeae* Falleroni, 1926, *Anopheles persiensis* Linton, Sedaghat and Harbach, 2003 and *Anopheles sacharovi* Favre, 1903 (Harbach 2004; Harbach 2015; Linton et al. 2007; White 1978).

At the territory of Serbia following AMC, species have been recorded so far: *An. atroparvus*, *An. labranchiae*, *An. maculipennis* s.s., *An. melanoon*, *An. messeae* and *An. sacharovi*, of which *An. atroparvus*, *An. maculipennis* s.s., *An. melanoon* and *An. messeae* are present in Vojvodina Province (north Serbia), formerly an area of widespread endemic malaria (Zgomba et al. 2002; Kostić 1946).

Malaria was a widespread disease in Europe until the second half of the twentieth century. Historic and also current endemic infections in Europe, particularly transmitted by *An. maculipennis* complex species, have been caused regularly by *Plasmodium vivax*, whose sporozoites readily develop also in temperate climates (Marí and Peydró 2012). However, there has been a substantial number of imported tropical malaria (*P. falciparum*) which accounts for about 77% of tropical disease cases in Europe (65,596 infections reported between 2000 and 2009, TropNetEurop 2010).

The high density of *Anopheles* species in many southern European regions (Romi et al. 1997; Ponçon et al. 2007; Marí and Peydró 2010) and the increasing importation of malaria infections in the last two decades have led to the reappearance of autochthonous malaria cases in Italy (Baldari et al. 1998), Greece (Kampen et al. 2002), France (Doudier et al. 2007) and Spain (Santa-Olalla Peralta et al. 2010). As a consequence of mass immigration and travel from malaria-endemic countries to Greece, 85 human malaria cases were recorded in 2015 (six locally acquired), 88 in 2016 (five locally acquired) and 75 in 2017 (five locally acquired) (HCDCP 2015, 2016, 2017). Recently, The Netherlands and France have reported malaria in the patients without any previous travel history (ECDC 2014, 2015).

After the discovery of *An. daciae* (Nicolescu et al. 2004), the new member of AMC, identification of complex members is based on the nucleotide sequence differentiation of the internal transcribed spacer 2 (ITS2) region of genomic rDNA genes. Sequence analyses reduced probable misidentification of AMC species, due to overlapping egg characters, in different geographic regions. Since then, the species of AMC were identified by ITS2 analyses in many countries: England (Linton et al. 2002; Danabalan et al. 2013), Greece (Linton et al. 2001; Patsoula et al. 2007), Germany (Proft et al. 1999; Weitzel et al. 2012; Kronefeld et al. 2012; Kronefeld et al. 2014), Iran (Sedaghat et al. 2003), Italy (Marinucci et al. 1999; Di Luca et al. 2004), Poland (Rydzanicz et al. 2017), Romania (Nicolescu et al. 2004) and Turkey (Simsek et al. 2011), but not in Serbia. Our study applies ITS2 sequence analyses for molecular *An. maculipennis* complex identification.

Serbia’s *Anopheles* fauna has been studied based on morphological characteristics of adults and eggs (Adamovic 1975a, b, c, 1982, 1983; Dakic et al. 2008). The main vector of malaria in Serbia (in the Belgrade region) was *An. maculipennis* s.s. Secondary vectors were *An. messeae* and *An. atroparvus* (Simic 1948; Vukasovic 1950; Sitar 1977). *Anopheles maculipennis* s.s. was the predominant anopheline mosquito in the hilly areas of the Vojvodina province (Adamovic 1982), while *An. messeae* was by far the most abundant in the villages near marshes in the alluvial plain of the Danube, Sava and Tisa rivers. A similar species distribution in comparable landscapes was found in Hungary, Romania and Germany (Weyer 1938).

Species records of *An. messeae* prior to 2003 most likely comprised *An. daciae*, the most similar sibling species. Considering the species identification according to egg shape and colouration, differences between *An. messae* and *An. daciae* are minor and statistically insignificant (i.e. to be outside the range of natural phenotypic variation within a species) (Nicolescu et al. 2004; Kronefeld et al. 2012; Jetten and Takken 1994; Hackett et al. 1932). Therefore, morphological identification methods cannot be considered as reliable.

Consequently, historical data about the distribution, ecology and malaria vector potential of *An. messeae* could be imprecise. According to recent studies, the ecological demands and the spatial distribution of both species seem to be widely overlapping (Weitzel et al. 2012; Danabalan et al. 2013; Kronefeld et al. 2014; Lühken et al. 2016). *An. atroparvus* was found as a predominant species in the areas of alkaline soils in East Vojvodina, particularly in the lowlands of the Tisa and Tamis rivers (Adamovic 1980).

The discovery of *An. daciae* in neighbouring Romania and Greece indicates the potential occurrence of the species in Serbia. Accordingly, studies on ecological characters such as primarily host and breeding site preferences could refer to both species to an unknown extent so far.

This study aims to assess the species occurrence, geographical distribution and abundance of *An. maculipennis* complex species and the degree of overlap of breeding site preferences of larval populations in light of the potential occurrence of *An. daciae* in northern Serbia. This is the first study in Serbia in which molecular identification was used to separate the species of *An. maculipennis* complex.

## Material and method

### Study area and mosquito collection

Mosquitoes were collected in the northern Serbian province Vojvodina, located in the lowest part of the Pannonian Plain. It has a total surface area of 21,500 km^2^, which accounts for 24% of Serbia’s territory. The mountains surrounding this lowland, mainly Fruska Gora (539 m) and Vrsac Hill (641 m), have a significant impact on its climate characteristics.

Vojvodina is rich in fertile loamy loess soil, brown forest soil with patches of alkaline soil and black hydromorphic mineral soil, which is periodically flooded by the Danube river and its tributaries. Alkaline soils were formed in shallow depressions of this area.

The climate of Vojvodina is moderate continental, with cold winters and hot and humid summers with a huge range of extreme temperatures and featuring inconsistent amounts of rainfall over the course of months, which led to different values of aridity types. The mean annual temperature is 11 °C, and the mean annual precipitation is 602 mm (Mihailovic et al. 2004).

The most capacious anopheline breeding sites are extensive oxbow marshes, swamps and old riverbeds in the alluvial plains of the rivers Sava, Tisa and Danube. They are characterised by permanently stagnant, fresh water and dense hydrophilic vegetation.

Mosquitoes were collected from the 30th of May until the 13th of August 2015. Adults were collected biweekly and larvae monthly. *An. maculipennis* complex species were separated through the morphological identification of all specimens (Becker et al. 2010; Schaffner et al. 2001).

**Fig. 1:**
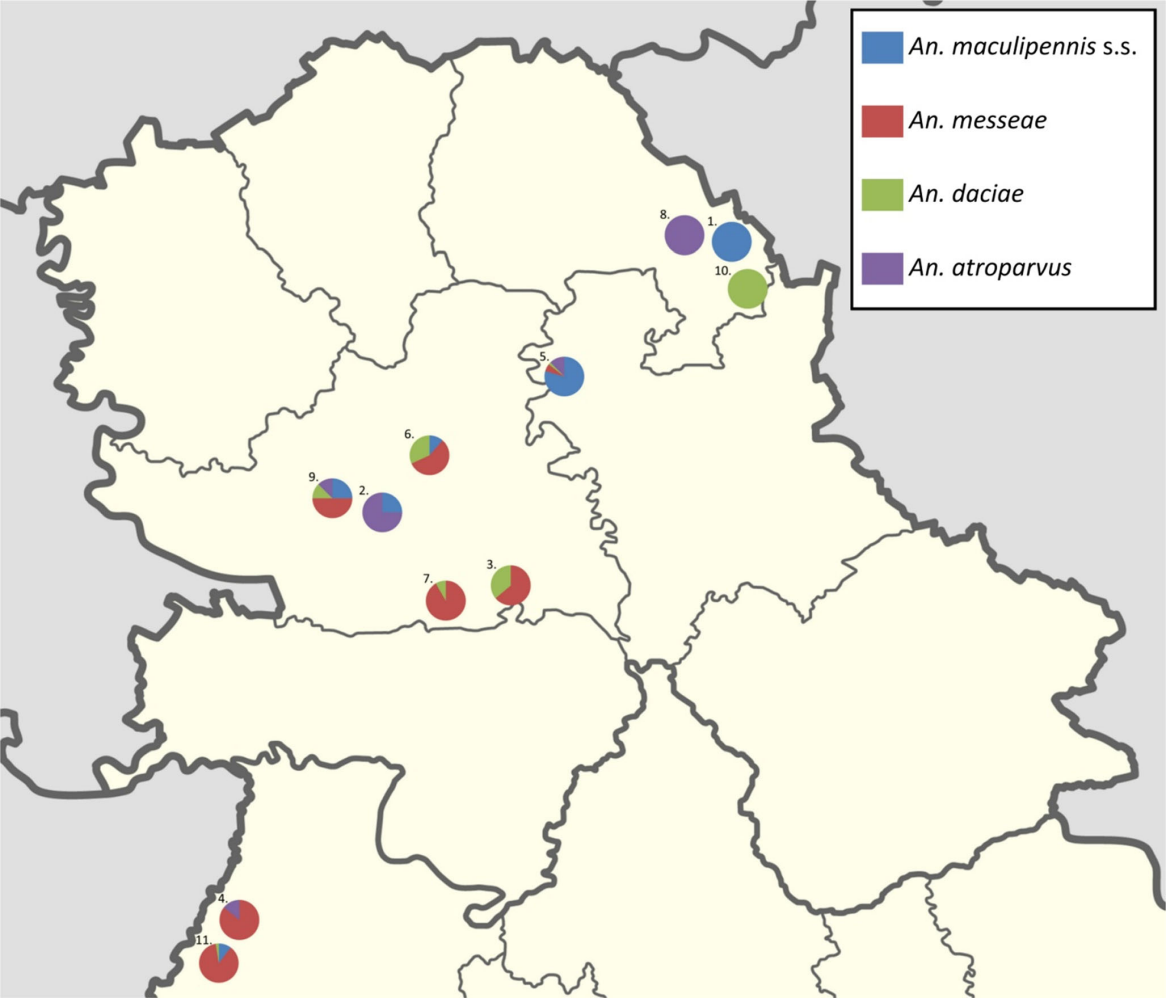
Records of *An. maculipennis* complex larvae in Vojvodina, Serbia

Larvae were sampled at 11 localities in rural and semi urban areas. In majority localities from one breeding site, but, at three of 11 localities, samples were taken from two breeding sites (Fig. 1, Table 1). Different types of larval breeding sites were chosen, as ditches, ponds, forest path filled with rainwater, swamps and marshes. The main criterion for the selection of sampling sites was the ecological diversity, aiming to determine the most attractive or typical for different *An. maculipennis* complex species. Larvae were collected by WHO dippers (350 ml volume); each sample consisted of ten dipps/breeding site. Larvae of L_2_, L_3_ and L_4_ instars were kept in 70% ethanol until molecular identification. Types and geographical coordinates of breeding sites, and species association indices are presented in Tables 1 and 2.

**Table 1:** Sampling locations and breeding sites in Vojvodina and respective species composition of 250 larval samples

No.	Locality	Geocoordinates	Breeding site	*mac* ^*1*^	*mess* ^*2*^	*dac* ^*3*^	*atr* ^*4*^	∑
1	Banatsko Veliko S.	45° 48′ 56″ N 20° 36′ 32″ E	Artificial lake	1	0	0	0	1
2	Kisač	45° 21′ 31″ N 19° 43′ 43″ E	Ditch	1	0	0	3	4
3	Kovilj	45° 13′ 28″ N 20° 1′ 9″ E	Ditch	0	23	13	0	36
4	Lešnica	44° 38′ 46″ N 19° 17′ 39″ E	Ditch	0	6	0	1	7
5	Novi Bečej	45° 35′ 40″ N 20° 8′ 33″ E	Ditch	42	6	0	4	52
5	Novi Bečej	45° 35′ 50″ N 20° 8′ 28″ E	Ditch	35	0	2	8	45
6	Sirig	45° 26′ 40″ N 19° 49′ 8″ E	Ditch	5	25	14	0	44
7	Petrovaradin	45° 15′ 16″ N 19° 53′ 35″ E	Forest path	0	11	1	0	12
8	Kikinda	45° 48′ 11″ N 20° 26′ 56″ E	Marsh	0	0	0	1	1
9	Bački Petrovac	45° 22′ 27″ N 19° 35′ 13″ E	Pond	0	1	0	0	1
10	Kozarci	45° 46′ 51″ N 20° 36′ 38″ E	Pond	0	0	1	0	1
11	Lipnički Šor	44° 35′ 41″ N 19° 14′ 1″ E	Pond	4	32	1	0	37
11	Lipnički Šor	44° 36′ 18″ N 19° 13′ 43″ E	Pond	0	2	0	0	2
9	Bački Petrovac	45° 21′ 6″ N 19° 35′ 41″ E	Swamp	2	3	1	1	7
Total				90	109	33	18	250

*mac*^1^ *An. maculipennis* s.s., *mess*^2^ *An. messeae*, *dac*^3^ *An. daciae*, *atr*^4^ *An. atroparvus*

**Table 2:** Species association indices (Dice 1945) based on the species occurrence in the eight most productive breeding sites

	*An. maculipennis* s.s.	*An. messeae*	*An. daciae*	*An. atroparvus*
*An. maculipennis* s.s.		0.57	0.67	0.75
*An. messeae*	0.80		0.83	0.75
*An. daciae*	0.80	0.71		0.50
*An. atroparvus*	0.60	0.43	0.33	
Breeding sites positive	5	7	6	4
Breeding site index	0.63	0.88	0.75	0.50
Avg. no. of *Anopheles* species per site	2.20	1.71	1.83	2.00

Two reciprocal values above and below the diagonal are obtained. Below, the number of positive breeding sites for the respective species, the breeding site index and the average number of *Anopheles* species in the respective breeding sites are quantified

**Fig. 2:**
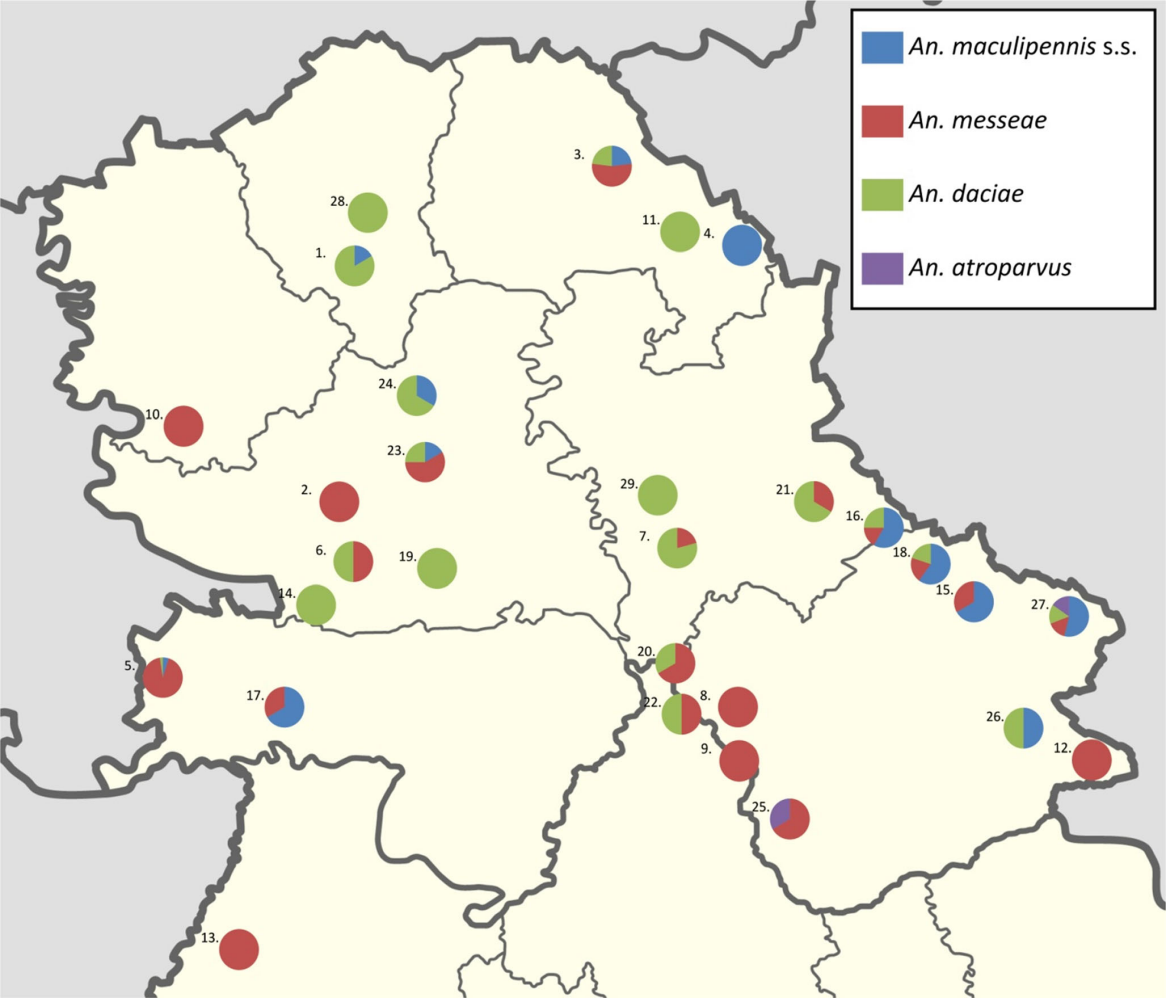
Distribution of the *An. maculipennis* complex adults in Vojvodina, Serbia

Adults were collected at 29 different urban and rural localities (Fig. 2, Table 3). The majority of localities (24) had only one collecting place. However, four localities had one additional collecting site and one location had three collecting sites. Adults were collected by CDC CO_2_ traps filled with dry ice (John W. Hock Company, Florida). Most of the traps were positioned near domestic animal stables, operating overnight. Traps were set up in the late afternoon and were collected in the morning. After morphological identification to the species complex level (Becker et al. 2010), all adults were conserved in vials containing 70% ethanol for molecular analysis.

**Table 3:** Sampling locations of adult trap collections in Vojvodina and respective species composition of 172 adult samples

Site no.	Locality	Collection place	Geocoordinates	*mac* ^*1*^	*mess* ^*2*^	*dac* ^*3*^	*atr* ^*4*^	∑ Total
1	Bačka Topola	Home garden	45° 49′ 33.77″ N 19° 37′ 57.41″ E	1	0	5	0	6
2	Bački Petrovac	Next to the pond	45° 21′ 5.48″ N 19° 35′ 41.47″ E	0	1	0	0	1
3	Banatski Monoštor	Henhouse	45° 57′ 25.78″ N 20° 16′ 41.16″ E	3	7	3	0	13
4	Banatsko Velikoselo	Pigsties, dog	45° 48′ 55.72″ N 20° 36′ 33.33″ E	2	0	0	0	2
5	Batrovci	Forest	45° 2′ 46.57″ N 19° 6′ 35.27″ E	2	39	1	0	42
6	Begeč	Home garden	45° 14′ 4.30″ N 19° 37′ 25.23″ E	0	0	2	0	2
	Begeč	Home garden	45° 14′ 0.55″ N 19° 37′ 39.48″ E	0	2	0	0	2
7	Ečka–Lukino Selo	Home garden	45° 18′ 39.55″ N 20° 25′ 23.01″ E	0	5	19	0	24
8	Glogonj	Home garden	44° 58′ 53.98″ N 20° 32′ 0.59″ E	0	1	0	0	1
9	Jabuka	Horse barn	44° 57′ 19.27″ N 20° 37′ 56.57″ E	0	1	0	0	1
	Jabuka	Home garden	44° 56′ 51.67″ N 20° 35′ 6.24″ E	0	1	0	0	1
10	Karavukovo	Home garden	45° 30′ 21.42″ N 19° 11′ 20.40″ E	0	1	0	0	1
11	Kikinda	Next to the pond	45° 48′ 8.99″ N 20° 26′ 50.36″ E	0	0	1	0	1
12	Kruščica	Henhouse	44° 55′ 22.84″ N 21° 27′ 58.91″ E	0	1	0	0	1
13	Lipnički Šor	Next to the pond	44° 35′ 41.80″ N 19° 14′ 0.11″ E	0	1	0	0	1
14	Lug	Henhouse	45° 11′ 15.26″ N 19° 32′ 35.07″ E	0	0	1	0	1
15	Margita	Henhouse	45° 12′ 52.25″ N 21° 10′ 42.36″ E	2	1	0	0	3
16	Markovićevo	Henhouse, dog	45° 19′ 29.62″ N 21° 1′ 59.76″ E	7	2	3	0	12
17	Martinci	Hen/goat/pigs house	45° 0′ 46.31″ N 19° 26′ 44.60″ E	2	1	0	0	3
18	Miletićevo	Goat pens, hens	45° 51′ 28.03″ N 19° 12′ 39.85″ E	0	1	0	0	1
	Miletićevo	Henhouse	45° 18′ 13.71″ N 21° 3′ 34.87″ E	3	0	1	0	4
19	Novi Sad	Home garden	45° 15′ 16.08″ N 19° 48′ 55.95″ E	0	0	1	0	1
20	Opovo	Home garden	45° 3′ 12.18″ N 20° 25′ 4.26″ E	0	1	0	0	1
	Opovo	Home garden	45° 0′ 59.41″ N 20° 28′ 21.58″ E	0	1	0	0	1
	Opovo	Home garden	45° 3′ 11.98″ N 20° 25′ 4.60″ E	0	0	1	0	1
21	Sečanj	Henhouse	45° 21′ 39.19″ N 20° 46′ 20.50″ E	0	1	0	0	1
	Sečanj	Henhouse	45° 21′ 59.92″ N 20° 46′ 21.04″ E	0	1	4	0	5
22	Sefkerin	Home garden	44° 59′ 4.64″ N 20° 31′ 18.69″ E	0	1	1	0	2
23	Sirig	Home garden	45° 26′ 41.82″ N 19° 48′ 58.92″ E	2	7	3	0	12
24	Srbobran	Home garden	45° 32′ 52.69″ N 19° 47′ 31.64″ E	1	0	2	0	3
25	Starčevo	Home garden	44° 49′ 14.67″ N 20° 43′ 40.75″ E	0	2	0	1	3
26	Straža	Henhouse, dog	44° 58′ 42.79″ N 21° 18′ 10.81″ E	1	0	1	0	2
27	Veliko Središte	Henhouse, rabbits	45° 11′ 48.49″ N 21° 23′ 44.01″ E	7	2	2	2	13
28	Zobnatica	Forest	45° 52′ 0.38″ N 19° 38′ 1.50″ E	0	0	3	0	3
29	Zrenjanin	Home garden	45° 21′ 33.00″ N 20° 23′ 29.77″ E	0	0	1	0	1
Total				33	81	55	3	172

*mac*^1^ *An. maculipennis* s.s., *mess*^2^ *An. messeae*, *dac*^3^ *An. daciae*, *atr*^4^: *An. atroparvus*

### DNA extraction and analyses

DNA was extracted individually from 422 mosquito samples using QuickExtract DNA Extraction Solution 1.0 (Biozym, Germany). According to the producer’s protocol, whole larvae or adults were homogenised using a pestle in a reaction tube before QuickExtract™ DNA Extraction Solution 1.0 was added. The volume of extract solution was 50 μL per adult and the same volume was used for the L_3_ and L_4_ larval stages. Lower amounts of solutions (25 μL) were used for 2nd instar larvae, respectively.

Smashed tissue was vortexed for 15 s, centrifuged for 1 min, incubated for 6 min at temp 65 °C and then vortexed again for 15 s, centrifuged for 1 min, incubated for 2 min at temp 98 °C and centrifuged for 1 min. Samples were stored in a freezer at − 20 °C until processed.

Standard PCR was carried out as described (Linton et al. 2001), utilising 5.8SF (5′-ATC ACT CGG CTC GTG GAT CG-3′) and 28SR primers (5′-ATG CTT AAA TTT AGG GGG TAG TC-3′) (VBC Biotech, Vienna, Austria) (Collins and Paskewitz 1996; Danabalan et al. 2013). PCR products were purified (PCR Clean Up extraction kit, GeneON, Germany) and then separated by 3% agarose gel electrophoresis (high resolution agarose, Roth, Germany), stained with Gelstar (Lonza, USA) and sized with Quantitas DNA low ladder (Biozym, Germany).

PCR products were further analysed by RFLP (*Bst*UI, New England BioLabs, Germany) (Danabalan et al. 2013). Different numbers of *Bst*UI recognition sites based on the diagnostic nucleotide sequence differences in the respective ITS2 regions permit the identification of *An. daciae*, *An. messeae/maculipennis* s.s. and *An. atroparvus* by diagnostic size and the number of fragments by 3% agarose gel electrophoresis (Danabalan et al. 2013).

Additionally, multiplex PCR (Kronefeld et al. 2014) was used to confirm the reliability of the PCR-RFLP assay for the differentiation of *An. messeae* from *An. maculipennis* s.s. The DNA of several samples was sequenced by Eurofins Medigenomix GmbH (Germany). DNA alignments were performed with CLC sequence viewer (CLC bio, Denmark; http://www.clcbio.com/products/clc-sequence-viewer) and BLAST (https://www.ebi.ac.uk/Tools/sss/ncbiblast/nucleotide.html).

Following the obtained results, species association indices were calculated by Dice (1945), based on the species occurrence in the eight most productive breeding sites (Table 2). Species association indices quantify the proportion of places where two species were found in combination compared to the total number of places where one of the respective species was found. From species association indices, the extent of overlapping demands on the biotic and abiotic structure of breeding sites may be estimated. Generally, more abundant and ubiquitous species may be found more frequently associated to others (Dice 1945).

#### Data availability

The datasets used and/or analysed during the current study are available from the corresponding author on reasonable request.

## Results

In 24 larval samples, 250 of them were successfully identified by molecular methods. Trap collections provided 47 samples with 172 adult specimens that were all morphologically identified to the species complex level and were analysed by PCR. Altogether, 422 specimens were successfully determined to the species level.

Larvae and adults of *An. daciae* were recorded for the first time in Serbia at 25 different locations in the Vojvodina province (Table 1, Table 3). In addition, three other *An. maculipennis* complex species were identified in different areas of the province: *An. maculipennis* s.s., *An. messeae* and *An. atroparvus* (Table 1, Table 3)*.* Single-species records (diagonal) and species-combined records (above the diagonal) at 29 localities in 47 adult trap collections are presented in Table 4.

**Table 4:** Single-species records (diagonal) and species-combined records (above the diagonal) at 29 geographical locations in 47 adult trap collections

	*An. maculipennis* s.s.	*An. messeae*	*An. daciae*	*An. atroparvus*
*An. maculipennis* s.s.	0	8	9	1
*An. messeae*		7	11	2
*An. daciae*			4	1
*An. atroparvus*				0

### Larval populations

In the collections of 250 larval specimens 109 (44%) *An. messeae*, 90 (36%) *An. maculipennis* s.s., 33 (13%) *An. daciae* and 18 (7%) *An. atroparvus* were identified (Table 1)*. An. messeae* and *An. maculipennis* s.s. were the predominant in all larval samples from 14 breeding sites and 11 geographic locations in Vojvodina (Table 1). Also, *An. daciae* was found widespread in various breeding sites but less numerous. *An. atroparvus* was least abundant and mostly found associated with other sibling species (Table 2)*.*

Each of the identified species was found in a variety of breeding sites at different geographical places across the region of Vojvodina.

Larvae of all species were found in each month during the sampling interval from the beginning of June to the middle of August 2015.

The distribution of all species within the complex was not only geographically overlapping, but the larvae of each species were found associated with other complex species in those breeding sites where numerous larvae could be collected (Table 2). *An. messeae* and *An. daciae* were very much connected to each other, and *An. maculipennis* s.s. was most universal, but was quantitatively dominant in ditches.

### Adult populations

**Fig. 3:**
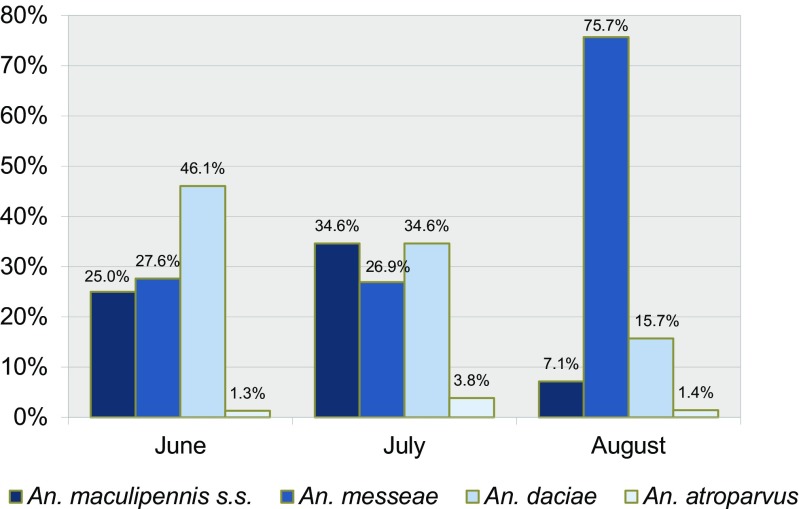
Species proportions of 47 adult samplings by CO_2_ traps during summer in the region Vojvodina, Serbia

In 47 trap collections of females at 29 locations, 81 *An*. *messeae*, 55 *An. daciae*, 33 *An. maculipennis* s.s. and 3 *An. atroparvus* were determined (Table 3). The average number of specimens per trap/night was 3.2 in June, 3.3 in July and 4.7 in August (Fig. 3).

Females of *An. messeae* were most widely distributed, found at 22 of 35 (66.7%) collecting places. The largest collection in one trap, 39 females/trap/night was also made for *An. messeae* in the forest region of Batrovci, indicating a considerable abundance. *An. daciae* was caught at 19 (57.6%) collecting sites and was the most abundant in Ecka–Lukino Selo, Banat village, close to Romania, where 13 females per trap/night were caught. *An. maculipennis* s.s. females were present in 14 (42.4%) collecting places by low numbers in various collections, whereas females of *An. atroparvus* were only present at two localities, Veliko Srediste and Starcevo village, with each one specimen per trap/night.

Females of *An. maculipennis* s.s. and *An. messeae* were included already in the first collections in May, whereas *An. daciae* and *An. atroparvus* were recorded in the traps in June (Fig. 3).

## Discussion

The study of various larval breeding sites and CO_2_-trap collections in the geographic region of Vojvodina, Serbia, gave a comprehensive insight into the species abundance in different areas, which were heavily infested by malaria until the mid-twentieth century. The recognition of *An. daciae* at various places and its occurrence in combination with other complex species complements and completes previous studies on the species composition and distribution of the *An. maculipennis* complex in Serbia. The finding of a wide overlap of breeding site specificity, particularly of *An. messeae* and *An. daciae*, supports the assumption that both species had previously been admixed and recorded together as *An. messeae*. Although *An*. *atroparvus* was described to occur mainly at coastal regions and in brackish waters (Adamovic 1983; Dakic et al. 2008; Vujic et al. 2010), this species could also be found associated with other complex species in the same breeding sites in Vojvodina, but not numerously. In particular, *An. messeae* could be found frequently in ponds filled up with clean water. Due to the size and availability of such waters in Vojvodina, it is not surprising that *An. messeae* is the most abundant species of the complex at many places. In contrast, *An. maculipennis* s.s*.* larvae were recorded predominantly in manmade or humanly affected breeding sites. Unselective choice of breeding sites and particularly the use of manmade, contaminated and also small waters may be crucial aspects in terms of association to livestock husbandry. Blood meals could be provided in traditional stables, serving as well-tempered refugium for oogenesis and simultaneously for the sporogonic cycle of *Plasmodium*, which made *An. maculipennis* s.s*.* the most capacious vector in wide parts of Serbia. During the last decades, some of these favourable conditions may have been reduced as a result of landscape management, structural changes in agriculture and human lifestyle. Overall, analysis of the species association in breeding sites revealed widely overlapping breeding site requirements of *An. maculipennis* s.s*.*, *An. messeae*, *An. daciae* and *An. atroparvus* that results in a wide geographic and spatial distribution of the four species, but with different abundance.

The CO_2_ trap collections conducted within this study confirmed the occurrence of the same four sibling species in Vojvodina. Only *An. atroparvus* was recorded very rare to receive sufficient geographic information. Close placement of the traps to livestock and stables revealed a variable connection of those species to such facilities. Compared to larval collections, adult *An. daciae* were more abundant than *An. maculipennis* s.s*.*, which could be a consequence caused by sampling variance, but also by a strong and unselective association of *An. daciae* to various animal stocks. Concerning seasonality, conclusions are very limited, except the finding that *An. messeae* records increased by season progression that could be in relation to the size of breeding sites combined with increased reproduction.

Dakic et al. (2008) collected adult mosquitoes inside animal shelters at eight different localities within the area of the Danube and Sava river basin near Belgrade (Serbia) between June and October in 2003. Females were determined by egg morphology, hence not discriminated between *An. messeae* and *An. daciae*. Three species of the *An. maculipennis* complex, *An. messeae*, *An. atroparvus* and *An. maculipennis* s.s., were identified. The most abundant species was *An. messeae* (64%, maybe including unrecognised *An. daciae* specimens), which is in accordance with the results of this study. The second most abundant species was *An. atroparvus*, with a proportion of 21%, and the least abundant species was *An. maculipennis* s. s. with 8%. *An. messeae* was equally prevalent in animal shelters with different animal hosts such as cows, pigs, sheep, goats and turkeys. It remains unclear to what extent the mode of sampling, in or close to animal shelters, is representative of the species abundance, since the species records might be influenced by host selectivity and specific habits of invasion into stables. *An. atroparvus* was very rare in animal shelters with different kinds of domestic animals, especially turkeys and goats, while *An. maculipennis* s.s. prefers animal shelters with cows and pigs (Dakic et al. 2008). The same authors described characteristics of breeding sites, where larvae of the *An. maculipennis* complex were sampled. *An. messeae* was mostly recorded in clean, alkaline waters (pH = 9), followed by fresh waters with a lower quantity of chloride (40–50 mg/L) and a minimum quantity of bicarbonate of 1 mg/L. The preference of *An. messeae* for clean waters was also confirmed by Weitzel et al. (2012). Furthermore, *An. atroparvus* preferred clean, alkaline water, but tolerated a greater quantity of chloride (60–90 mg/L) and bicarbonate (500 mg/L). *An. maculipennis* s.s. was mainly found in waters with higher quantity of ammonia (47.44 mg/L) and mud, which is in accordance with the findings of this species predominantly in manmade and contaminated ditches (Dakic et al. 2008).

Adamovic (1982) studied *Anopheles* species in Srem, Vojvodina, which was according to Simic (1957) region of endemic malaria. He found *An. maculipennis* s.s. at all examined sites in a range from 11.8 to 95.9% and *An. messeae* with a slightly lower relative proportion of 4.1 to 86.7%. *An. maculipennis* s.s. was also common in our study, but *An. messeae* was more abundant, especially in adult trap collections on the territory of Vojvodina. The present results of *An. atroparvus* larval collections are in accordance with adult abundance obtained in the traps of this study. Species was rare sampled in both stages (larvae and adults). However, *An. atroparvus* was earlier found in low numbers, but widespread at 7 of 11 examined sites (Adamovic 1982).

In previous papers by Guelmino et al. (1951) and Adamovic (1975a), *An. atroparvus* was recorded as the predominant species near Smederevo and in the north part of the lowland of the Tisa river in the Pannonian Plain. They concluded that water management and drainage of wetlands during the second half of the twentieth century had impact on the abundance of *An. atroparvus*.

*An. daciae* is present in Germany (Kronefeld et al. 2012; Weitzel et al. 2012) and widely distributed (Kronefeld et al. 2014). In some regions of Wales and England, it was found more abundant than *An. messeae* (Danabalan et al. 2013), which was not the case in our study area.

Novikov and Vaulin (2014) found *An. messeae*/*An. daciae* to be the most widely distributed in European Russia and also most associated with other species, as *An. maculipennis* s.s. and *An. beklemishevi.* They concluded that combinations of coinhabiting species indicate the widest ecological niche of *An. messeae*/*An. daciae* compared to the other two.

## Conclusion

Although malaria’s receptivity is still high in different parts of Europe, Marí and Peydró (2012) concluded that the malariogenic potential for endemic transmission is low. More attention should be paid to the increasing trend of malaria importation due to the increase of tourist and refugees mobility. Corresponding prophylactic measures during their travels to/from endemic areas should be taken in consideration.

Laboratory tests carried out on European populations of the *An. maculipennis* complex demonstrated that *An. atroparvus* can transmit Asian strains of *P. vivax* and African strains of *P. ovale* but is refractory to African strains of *P. falciparum* (James et al. 1932; Garnham et al. 1954; Ramsdale and Coluzzi 1975; Ribeiro et al. 1989; Teodorescu 1983).

In the present study, four sibling species of the *An. maculipennis* complex were identified on the territory of Vojvodina: *An. messeae*, *An. maculipennis* s.s., *An. daciae* and *An. atroparvus.* The newly described species *An. daciae* could be found both as larvae and as adults for the first time in Serbia.

*An. daciae* was differentiated from *An. messeae* for the first time in the region of Vojvodina in this study. Both species have a sympatric distribution in Europe so far. Due to limited access to the molecular tools for species differentiation, *An. daciae* was inevitably overseen. Consequently, potential *An. daciae* specimens have been most likely recorded as *An. messeae* in all studies prior to 2004. Thus, ecological, biogeographical and epidemiological data established for *An. messeae* in the past have to be attributed to either one or both species. This study contributes to a new assessment of *An. maculipennis* complex species distribution and abundance in Vojvodina. Besides abundance and geographic distribution of *An*. *daciae* records, changes in the abundance of all complex species in comparison to historical data are also very important and require further, complex assessment. At present, the most abundant species in Vojvodina is *An. messeae*. In order to estimate vector competence, *An. daciae* should be tested to its susceptibility to *Plasmodium*. Marí and Peydró (2012) believe that *An. daciae*, as a recently described species of *An. maculipennis* complex, could had played a role in malaria transmission earlier, but attributed as vector was *An. messeae.* As *An. daciae* is widespread in eastern Europe, our study demonstrates that the Balkan countries should be included in the area of its distribution.
